# Whole exome sequencing of paediatric patients with Cogan’s syndrome to identify monogenic mimics

**DOI:** 10.1093/rheumatology/keag436

**Published:** 2026-08-25

**Authors:** Kirsty McLellan, Fiona Price-Kuehne, Alice Burleigh, Neil Martin, Sarah Hicks, Lisa Hutton, Sandrine Compeyrot-Lacassagne, Despina Eleftheriou, Ebun Omoyinmi, Paul Brogan

**Affiliations:** Infection, Immunity and Inflammation, University College London Great Ormond Street Institute of Child Health, London, UK; Paediatric Rheumatology, Royal Hospital for Children, Glasgow, UK; Infection, Immunity and Inflammation, University College London Great Ormond Street Institute of Child Health, London, UK; Infection, Immunity and Inflammation, University College London Great Ormond Street Institute of Child Health, London, UK; Paediatric Rheumatology, Royal Hospital for Children, Glasgow, UK; Paediatrics, Raigmore Hospital, Inverness, UK; Paediatrics, Raigmore Hospital, Inverness, UK; Rheumatology, Inverclyde Royal Hospital, Greenock, UK; Infection, Immunity and Inflammation, University College London Great Ormond Street Institute of Child Health, London, UK; Paediatric Rheumatology, Great Ormond Street Hospital for Children NHS Foundation Trust, London, UK; Infection, Immunity and Inflammation, University College London Great Ormond Street Institute of Child Health, London, UK; Paediatric Rheumatology, Great Ormond Street Hospital for Children NHS Foundation Trust, London, UK; Infection, Immunity and Inflammation, University College London Great Ormond Street Institute of Child Health, London, UK; Infection, Immunity and Inflammation, University College London Great Ormond Street Institute of Child Health, London, UK; Paediatric Rheumatology, Great Ormond Street Hospital for Children NHS Foundation Trust, London, UK

**Keywords:** Cogan’s syndrome, hearing loss, vasculitis, whole exome sequencing, monogenic mimic

## Abstract

**Objectives:**

Cogan’s syndrome (CS) is a rare variable vessel vasculitis, describing sensorineural hearing loss (SNHL), inflammatory ocular disease and vestibular dysfunction. We hypothesized that within paediatric-onset (p)CS, a proportion would have monogenic disease, either autoinflammatory and/or associated with SNHL.

**Methods:**

Whole exome sequencing (WES) was performed and analysed using an in-house pipeline incorporating virtual gene panels for inflammation and SNHL; copy number variant analysis (ExomeDepth); and phenotype-driven variant prioritization (Exomiser). Genetic variants were interpreted by a multi-disciplinary team according to American College of Medical Genetics and Genomics guidelines.

**Results:**

Ten patients with a clinical diagnosis of pCS were enrolled. Three/10 (30%) had a monogenic contribution to the phenotype based on Class 4/5 variants: *de novo NLRP3* p.T915R (*n* = 1) associated with Cryopyrin-associated periodic syndrome; *MYO7A* p.K542Qfs*5 (*n* = 1) causing SNHL; and *HBB* homozygous p.E7V causing sickle cell disease (associated with hearing loss and uveitis). A further two cases had possible monogenic contribution with the following rare variants of uncertain significance (class 3): *ADGRV1* compound heterozygous variants (*n* = 1) associated with Usher syndrome; and a novel *ALPK1* p.H735P (*n* = 1), associated with Retinal dystrophy Optic nerve oedema Splenomegaly Anhidrosis Headache (ROSAH) syndrome.

**Conclusions:**

In children presenting with features suggesting CS, genetic screening should be considered before conferring this rare diagnostic label since at least 30% had an alternative monogenic contribution to the phenotype rather than true pCS, with implications for treatment and prognosis. We thus advocate for genetic testing using next-generation sequencing for patients presenting with pCS.

Rheumatology key messages

- Monogenic autoinflammatory conditions mimicking Cogan’s syndrome were identified in 3/10, altering management and prognosis.
- Non-inflammatory monogenic disease, including non-syndromic hearing loss may contribute to the phenotype.
- Genetic testing should be considered for children presenting with suspected Cogan’s syndrome.

## Introduction

Cogan’s syndrome (CS) is a rare vasculitis affecting paediatric and adult patients. Typical cases are characterized by ocular, auditory and vestibular involvement; with typical defined by presence of interstitial keratitis, subconjunctival haemorrhage, conjunctivitis or iritis, with audio-vestibular involvement within 2 years of ocular involvement. Atypical forms are defined by presence of other ocular inflammatory patterns, vestibular involvement not Menières-like, or if audio-vestibular involvement occurs over 2 years later than ocular involvement (Table 1) [1–3]. Typically ocular and auditory symptoms develop simultaneously or sequentially within 1–2 years, and are often bilateral [4]. Systemic involvement occurs in 50–80% of cases [2, 5] and vasculitis can affect small, medium or large vessels, including the aortic root in 10% [4]. There is a recognized association between atypical CS and tubulo-interstitial nephritis and uveitis (TINU) [6]. The incidence increases in the second to fourth decades, but there is no sex or ethnic association [2, 7]. The aetiology of CS remains unknown. Based on current evidence CS is most likely a polygenic autoimmune-driven disorder, although there are no reported Human Leucocyte Antigen (HLA)-genotype associations [5]. Despite treatment with glucocorticoids, usually combined with immunosuppressive medications, sensorineural hearing loss (SNHL) is severe and irreversible in 50–90% of patients, necessitating cochlear implantation with early implantation recommended due to poorer outcomes associated with complete or partial intracochlear duct neo-ossification [2, 4].

**Table 1:** Clinical features of Cogan’s syndrome, modified from Tayer-Shifman *et al*. [3].

System	Clinical manifestations
Auditory	Sensorineural hearing loss
Ocular (defined as atypical if ocular involvement does not involve interstitial keratitis, subconjunctival haemorrhage, conjunctivitis or iritis)	Interstitial keratitis (classically) Uveitis (most common in clinical practice) Subconjunctival haemorrhage Conjunctivitis Scleritis Papilloedema Optic neuritis Central vein occlusion Glaucoma Retinal vasculitis
Vestibular/neurological (defined as atypical if symptoms not like Menières or if auditory involvement >2 years after ocular)	Menières-like attacks, loss of balance, vertigo, nausea, vomiting, tinnitus
Systemic	Lymphadenopathy Raised inflammatory markers Constitutional symptoms Arthralgia
Vascular	Vasculitis (classically large vessel)

We aimed to describe the phenotype of a cohort of paediatric-onset (p)CS in the UK and hypothesized that there would be a subgroup with a monogenic cause resulting in, or contributing to, the CS phenotype. This is observed in other vasculitides including polyarteritis nodosa, and Behçet’s disease, the latter now known to have several monogenic mimics [8, 9]. If the same was true of CS, this could have profound impact on the management and prognosis of the condition, and genetic counselling of patients. The aims of this study were therefore to describe a cohort of pCS in the UK and, using whole exome sequencing (WES) specifically explore if any had a monogenic autoinflammatory disease and/or a monogenic cause of non-syndromic SNHL resulting in the CS phenotype.

## Methods

### Patients

Patients with a clinical diagnosis of typical or atypical CS, based on the local senior clinician managing the case, were recruited from Great Ormond Street Hospital for Children, London or the Scottish Paediatric and Adolescent Rheumatology Network. SNHL was the primary criterion, with patients with inflammatory eye disease or features in keeping with typical or atypical CS eligible for inclusion. Patients or their legal guardian (if < 16 years) provided written consent (and assent if appropriate) with ethical approval in place (reference 08/H0713/82). Clinical data were extracted from electronic patient records and data analyses were descriptive, presented using median and interquartile range (IQR).

### Ethics

Ethical approval was obtained from the National Research Ethics Service, Bloomsbury Committee (08/H0713/82). All adult subjects provided written informed consent to participate; parental/legal guardian consent was obtained for all children with age-appropriate consent/assent as required.

### Genomic analyses

**Figure 1:**
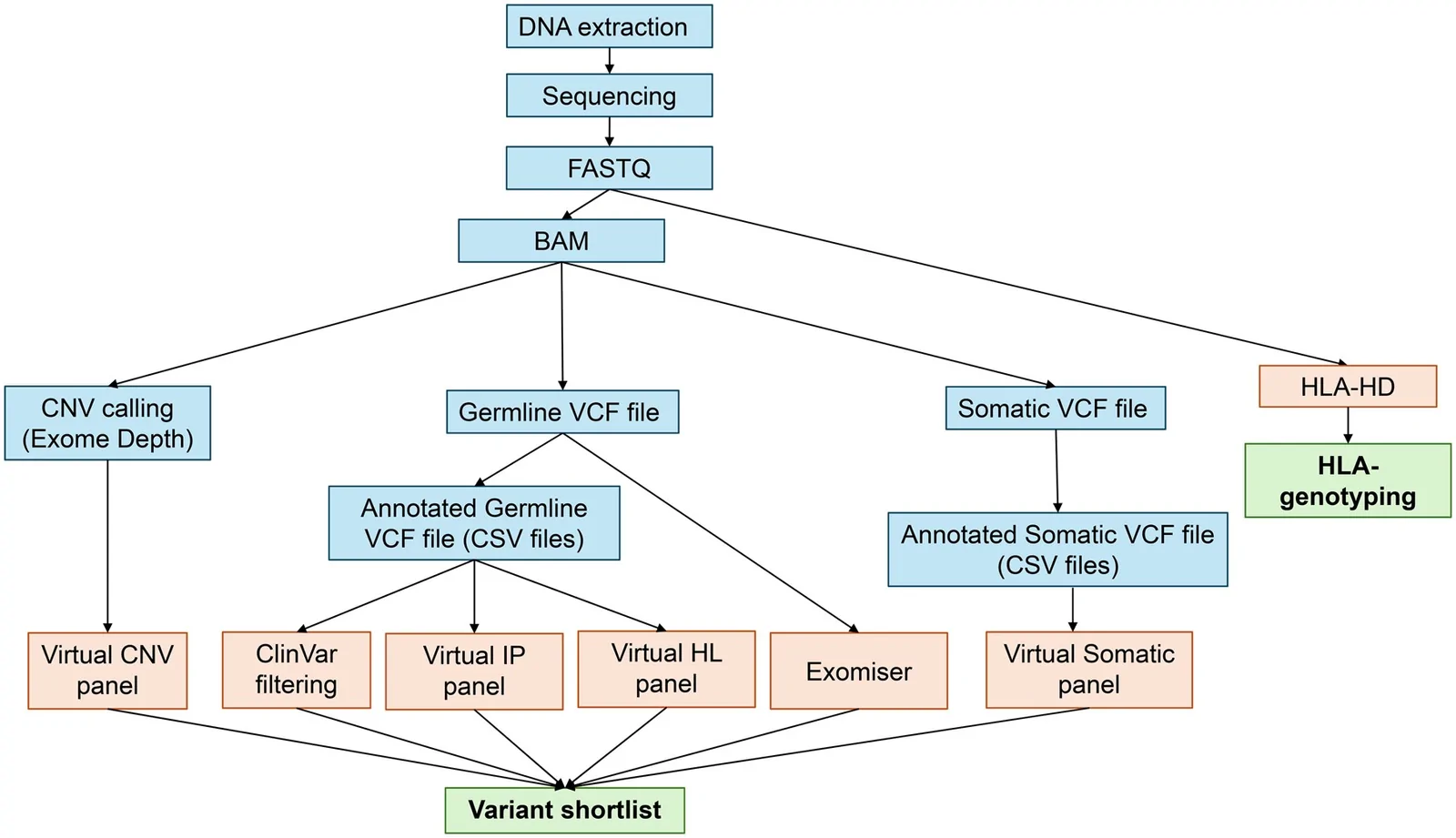
The bioinformatic pipeline for germline and somatic variants used to generate a variant shortlist for each participant. BAM, binary alignment map; CNV, copy number variant; CSV, comma separated value; HL, Hearing Loss; IP, inflammatory panel; VCF, variant call format

Patient DNA was either obtained from the referring clinical genetic laboratory (*n* = 9) or extracted from saliva using prep IT.L2P (DNA Genotek Inc.) (*n* = 1). WES was performed, using Illumina sequencing, with alignment and variant calling also performed by Informed Genomics Ltd using their ExomeCG Cells3™ service (https://nonacus.com/exome-cg/) as previously described [8] and the resulting variant call format (VCF) files annotated with ANNOVAR [10]. Variants were then filtered using a multi-pronged in-house R pipeline designed to both identify known monogenic diagnoses and facilitate novel discoveries, as per Fig. 1. Synonymous variants and variants with mean allele frequency (MAF) > 1% in GnomAD (https://gnomad.broadinstitute.org), ExAC [11] and G1000 (https://www.internationalgenome.org) were excluded, then variants with an existing assertion of pathogenic/likely pathogenic on ClinVar were scrutinized. Next, a hearing loss gene panel consisting of *n* = 154 genes was applied (Supplementary Table S1) designed using SNHL genes from PanelApp (https://panelapp.genomicsengland.co.uk) and the Hereditary Hearing loss database (https://hereditaryhearingloss.org/), in addition to a larger panel of 786 genes associated with inflammation/vasculitis [12, 13]. Somatic variants in *NLRC4*, *NLRP3*, *NOD2*, *STING1*, *TNFRSF1A* and *UBA1* were also assessed, since these can be pathogenic and of potential relevance to the CS phenotype. Genes affected by copy number variation identified by ExomeDepth were also assessed by filtering against the hearing loss and inflammation/vasculitis virtual panels [14]. Finally, the phenotype-driven tool Exomiser [15] was applied, with clinical information encoded using Human Phenotype Ontology terms (https://hpo.jax.org) and variants with Exomiser score >0.7 shortlisted. Variants of uncertain significance were additionally reviewed using *in silico* prediction scores, including AlphaMissense, a deep-learning model for pathogenicity prediction based on AlphaFold [16]. The variant shortlist generated for each patient was then discussed in a multi-disciplinary team (MDT) meeting consisting of Paediatric Rheumatology Consultants (P.B., D.E., K.M.), Clinician Scientist (F.P.-K.) and Genetic Scientists (A.B., E.O.) to assess the relevance of the variants to the reported phenotype and assign pathogenicity classes according to the American College of Medical Genetics and Genomics (ACMG) guidelines [17].

## Results

### Demographics and phenotype

Ten patients with a diagnosis of pCS were recruited. Clinical features, investigations and treatment are summarized in Table 2. Five/10 (50%) patients were female. The median age at symptom onset was 12.8 years (interquartile range (IQR) 9.2, 14.3) and median age at clinical presentation 13.4 years (IQR 9.8, 15.3). Four/10 (40%) were of Black African ancestry, 2/10 (20%) White British, 1 (10%) mixed Asian, 1 (10%) mixed White and Black African, 1 (10%) Other Black and 1 (10%) Arab ancestry, as per NHS Ethnicity codes. One patient (10%) had a background of consanguinity and 1 was from a close ancestral group. Nine/10 (90%) had ocular involvement; 8 of whom had bilateral involvement. Ten/10 (100%) had auditory involvement, 9 with bilateral sensorineural hearing loss, 1 with reduced cochlear hair cell function although normal hearing to date. Ocular involvement usually preceded auditory involvement; median time between symptom onset and ocular involvement was 0 (IQR 0,1) months and symptom onset to auditory involvement 3.6 (IQR 1.5, 5.7) months. Nine/10 (90%) had vestibular involvement. Seven/10 (70%) had neurological involvement, all with headache; 4/10 (40%) had renal involvement; 1/10 (10%) large vessel vasculitis; and 8/10 (80%) had systemic involvement. All are alive at last follow-up (median follow-up 74 months).

**Table 2:** Paediatric Cogan’s syndrome: clinical and laboratory characteristics.

Case	Sex	Age at first symptom onset (years)	Age at presentation (y)	Ethnicity	Ocular involvement	Auditory/vestibular involvement	Other systems involved	Investigations	Treatment	Outcome at last follow-up (FU) (duration of follow-up (months)
1	F	5.8	6.2	Black African	Bilateral uveitis, later developed optic disc oedema and papilloedema and posterior synechiae	Bilateral progressive complete hearing loss and vestibular symptoms with unsteadiness	Intermittent headaches	Normal CRP/ESR SAA elevated (156 mg/l; normal range <10) Mildly elevated urine NAG/creatinine ratio (31 U/g creatinine; normal range 2–12) MRI ears: normal CT ears: normal MRI head: labyrinthitis, papillitis, uveitis CT head: normal	Current: Methotrexate Adalimumab Previous: Corticosteroids (8 months)	19 months FU Bilateral cochlear implantation, papilloedema and raised ICP at 13 months post-diagnosis
2	M	9.1	10.3	White British	Bilateral episcleritis, later developed optic neuritis and papilloedema	Bilateral hearing loss Vestibular symptoms with rotatory vertigo	Fatigue, arthritis and nosebleeds Headaches Absence of urticarial-like rash	CRP elevated (25 mg/l; max 89 mg/l, normal range 0–10). Transient rise in creatinine (max 89 µmol/l) 8 months post-diagnosis. MRI: opacified mastoid air cells, ears normal MRI head: normal Lumbar puncture: elevated opening pressure and CSF pleocytosis (see main text)	Current: Tocilizumab Previous: Methotrexate Mycophenolate Mofetil Infliximab Corticosteroids (intermittent courses over 20 months)	41 months FU Developed papilloedema with raised ICP 24 months following dx
3	M	15.7	16.2	Black African	Bilateral uveitis, later developed posterior synechiae	Bilateral hearing loss, with auditory distortion Dizziness and nausea	No systemic symptoms Hepatic steatosis	Normal inflammatory markers ANA 1:160 Mild renal dysfunction (GFR 77 ml/min/1.73 m^2^) and mildly elevated renal tubular proteins (urine NAG/creatinine ratio 17 U/g creatinine, Urine RBP 35.8 mg/l (normal range 3.9–32)) MRI ears/head: vascular loop of anterior inferior cerebellar artery, some thickening of anterior ethmoid sinuses. CT head: slightly dysmorphic upper part of the cochlear CXR: Bilateral pleural effusions CT chest: normal Echocardiography: mildly dilated ventricular cavity MRA cardiac/whole body: normal	Current: MMF Adalimumab Previous: Corticosteroids (10 months)	21 months FU Developed early lens opacity at 18 months post-diagnosis
4	F	14.7	15.4	Black African	Unilateral uveitis	Bilateral hearing loss Light-headedness and nausea	Fever, weight loss, reduced appetite Headaches, forgetfulness & localized hypoaesthesia Renal involvement with renal impairment, proteinuria, haematuria: diagnosed with tubule-interstitial nephritis	Renal: raised creatinine (214 µmol/l), proteinuria (urine ACR 23.5 mg/mmol) (normal range 0.1–7.4); raised tubular proteins; urine NAG/creatinine ratio 176 U/g creatinine, Urine RBP 12671 mg/l Renal biopsy: immune-mediated glomerulonephritis Raised inflammatory markers (CRP 37 mg/l, ESR >120 mm/h (normal range 0–10)) Positive ANA (1:640; increased 1:2560) MRI ears: normal MRI head: multiple, small, non-specific, longstanding white matter changes, without enhancement	Current: MMF Previous: Corticosteroids (2 months) Methotrexate	6 months FU Unilateral cochlear implantation
5	M	12.9	13.0	Black African	Bilateral uveitis, retinal vasculitis. Papilloedema Later developed posterior synechiae	Reduced cochlear hair cell function, normal hearing Left lateral semicircular canal hypofunction Dizziness, nausea and loss of balance	Fatigue, weight loss, reduced appetite, abdominal pain Headaches Renal involvement: renal impairment, proteinuria. Diagnosed tubule-interstitial nephritis	Elevated inflammatory markers (CRP 55 mg/l, ESR 99 mm/h) Elevated creatinine (118 µmol/l at diagnosis, max 141). Proteinuria (PCR 93.8 mg/mmol) (normal range <20)) Raised tubular proteins; urine NAG/creatinine ratio 127 U/g creatinine, Urine RBP 1043 mg/l MRI ears: lack of signal in posterior branch of the left lateral semicircular canal CT: inflammatory diverticulum in semicircular canal MRI head: raised intracranial pressure, partially ossified left lateral semi-circular canal, possible labyrinthitis ossificans	Current: MMF Infliximab Previous: Methotrexate Adalimumab Corticosteroids 12 months	38 months FU Staph aureus left lower leg cellulitis/abscesat 30 months post-dx
6	F	13.6	13.8		Bilateral uveitis, later developed papilloedema	Bilateral hearing loss Vertigo	Fever Arthralgia Rash Headaches Renal: raised renal tubular markers; diagnosed tubule-interstitial nephritis.	Raised inflammatory markers (CRP 30 mg/l at diagnosis, max 51, ESR 45 mm/h) Mild renal impairment (creatinine 98 µmol/l) Raised tubular proteins; urine NAG/creatinine ratio 30 U/g creatinine, Urine RBP 301 mg/l Renal biopsy: patchy tubulo-interstitial nephritis. Skin biopsy: mild perivascular lymphocytic infiltrate. MRI ears/head: normal Echo: small pericardial effusion	Current: MMF Previous: Corticosteroids (8 months)	52 months FU Unilateral cochlear transplantation Papilloedema and raised ICP at 20 months Peripapillary fibrosis
7	F	12.8	15.4	Middle Eastern	Bilateral uveitis	Bilateral hearing loss Tinnitus, nausea, dizziness and vomiting	Weight loss, fatigue. Headache Renal: mild renal impairment and proteinuria at presentation	Normal CRP, mildly elevated ESR (28 mm/h) Renal: mildly raised creatinine initially, proteinuria (1 g in 24 h collection) MRI ears/head: normal	Current: None Previous: Corticosteroids (3 months)	33 months FU Bilateral cochlear implantation
8	M	14.5	14.8	White British	Bilateral interstitial keratitis, anterior stromal keratitis, stromal opacities, posterior stromal abnormalities, uveitis, iritis	Bilateral hearing loss, auditory distortion Tinnitus Vertigo, reduced balance	Lethargy	Normal inflammatory markers MRI ears/head: small amount of fluid in right mastoid air cells, otherwise normal	Current: Methotrexate Rituximab Previous: Corticosteroids (intermittent over 24 months) Adalimumab Tocilizumab	59 months FU
9	M	9.3	9.6	Mixed Asian	None	Bilateral hearing loss Tinnitus	Large vessel vasculitis, with widespread aneurysms Reduced appetite, weight loss, mouth ulcers Bilateral nephrectomies for widespread aneurysms No intrinsic renal disease	Elevated ESR (90 mm/h), SAA (973 mg/l), normal CRP. MRI head: intracranial aneurysms CT ears: normal Echocardiogram: normal MRA whole body: severe multifocal aneurysms affecting all arterial beds, severe abdominal aneurysm, bilateral severe renal artery aneurysms	Current: Corticosteroids MMF Adalimumab Tacrolimus Previous: Azathioprine Infliximab Cyclophosphamide	26 months FU Bilateral cochlear implantation Bilateral nephrectomies due to bilateral severe aneurysms, renal transplantation, sepsis, post-transplantation lymphoproliferative disease
10	F	6.5	7.3	Mixed White British/Black African	Bilateral uveitis	Bilateral hearing loss Unsteadiness and loss of balance	Lymphadenopathy Headaches Sickle cell anaemia	Elevated CRP 136 mg/l; max 187, with normal ESR MRI ears: bilateral labyrinthitis ossificans involving semicircular canals, loss of normal endolymph signal in semicircular canals. Suggestion of min thrombus within the cochlear arteries which would indicate vascular occlusion CT ears: bilateral labyrinthitis ossificans involving semicircular canals MRI head: normal CXR: subsegmental collapse	Current: None Previous: Corticosteroids (5 months)	129 months FU Bilateral cochlear implantation

ACR, albumin: creatinine ratio; ANA, anti-nuclear antibody; CRP, C-reactive protein; CXR, chest X-ray; ESR, erythrocyte sedimentation rate; F, female; GFR, glomerular filtration rate; ICP, intra-cranial pressure; M, male; MRA, magnetic resonance angiography; PCR, protein:creatinine ratio; RBP, retinol binding protein; SAA, serum amyloid A; urine NAG/creatinine ratio, urine N-acetyl-β-d-glucosaminidase/creatinine ratio.

### Investigations and treatment

Investigations (including imaging and biopsy results, where available) and treatment for each case are summarized in Table 2. Nine/10 patients had audio-vestibular magnetic resonance imaging (MRI) of the head/auditory system: 3/9 (33%) had audio-vestibular abnormalities; 1 showing labyrinthitis; 1 possible labyrinthitis ossificans and an inflammatory diverticulum; 1 with labyrinthitis ossificans and probable cochlear artery thrombus. One further patient had non-specific white matter changes on MRI head; and another had intracranial aneurysms in the context of widespread aneurysms. One patient had a dysmorphic appearance of the cochlear detected on CT scan. Nine/10 patients had serum angiotensin-converting enzyme measured at diagnosis, all of which were normal (not done for case 7).

Treatment for each case is shown in Table 2. All 10 (100%) patients received glucocorticoids; median duration of corticosteroids was 11.0 months (IQR 5.8,15.0). Two of 10 (20%) patients were managed with glucocorticoids alone, and both subsequently had cochlear implantation. The remaining eight cases were treated with immunosuppressive medications and remain on these at last follow-up.

### Outcome

All patients were alive at the end of follow-up. Nine/10 (90%) had hearing loss; 5/9 with hearing loss (56%) managed with cochlear implantation. Four/10 (40%) patients developed papilloedema and raised intracranial pressure, 2 treated with acetazolamide. One/10 (10%) developed cataract. One/10 (10%) had *Staphylococcus aureus* lower leg cellulitis. One patient required emergency bilateral nephrectomies, haemodialysis and subsequent renal transplantation secondary to severe (rupturing) aortic aneurysm associated with LVV (no evidence of intrinsic renal disease), an episode of severe sepsis; and subsequently developed an EBV-driven smooth muscle malignancy secondary to immunosuppression.

### Genomic analyses

WES was carried out on all 10 patients. A rare genetic variant which could fully explain or contribute to the phenotype was found in 5 patients, as shown in Table 3.

**Table 3:** Variants of significance identified in the pCS cohort.

Case	Gene (transcript)	Variant (Zygosity)	Associated disease (Inheritance)	MAF as per population databases: gnomAD ExAC G1000	*In silico* predictors (SIFT/LRT/Mutation-Taster)	Classification (ACMG criteria)**	Clinical interpretation
1	No genotype to explain the phenotype						
2	*NLRP3* (NM_004895)	c.C2744G p.(T915R) (het)	CAPS (AD)	0 0 0	T/N/N	4 (PS2 (*de novo* in a patient with disease and no family history) and PM2 (absent from controls in population databases))	Novel *de novo* variant in *NLRP3*, gene associated with CAPS. This variant was identified on the IP and HL virtual gene panels and Exomiser.
3	*ALPK1* (NM_025144)	c.A2204C p.(H735P) (het)	ROSAH syndrome (AD)	0 0 0	T/N/N	3 (PM2 (absent from controls in population databases))	Novel variant in *ALPK1* associated with ROSAH syndrome. Identified on the IP panel.
4	*ADGRV1* (NM_032119)	c.G1849A p.(V617M) (het) c.G3289A p.(G1097S) (het) c.A6994T p.(I2332F) (het)	Usher syndrome (AR)	0.0003 0.0002 0.000599 0.0053 0.0045 0.003994 0.0003 0.0002 0.000599	D/N/D D/D/D D/D/D	All variants: 3 (PP3 (computational evidence supports a damaging effect on the gene)	A previous report of an individual with compound heterozygous variants (c.G1849A & c.A6994T) in an individual with Usher syndrome type 2C. Unable to ascertain from our workflow if variants are *cis* or *trans* and therefore parental studies would be next line of investigation.
5	No genotype to explain the phenotype						
6	No genotype to explain the phenotype						
7	No genotype to explain the phenotype						
8	*MYO7A* (NM_000260)	c.1617dupC p.(K542Qfs*5) (het)	AD deafness (AD)	1.22E-05 0.0001 0	././.	5 (PVS1 (Null variant leading to premature translational stop signal with loss of functional recognized mechanism of disease), PS4 (prevalence in affected individuals higher than in controls) and PM2 (not present in controls))	This variant is previously documented in heterozygous state as the cause of a hearing loss syndrome and two reports of this variant, in heterozygous state, along with an additional *MYO5A* variant in the context of Usher syndrome.
9	No genotype to explain the phenotype						
10	*HBB* (NM_000518)	c.A20T p.(Glu7Val) (hom)	Sickle cell anaemia (AR)	0.0034 (African 0.0478) 0.0044 (African 0.0485)	D/./N	5 (PS1 (same amino acid change as a previously confirmed variant), PS3 (deleterious effect on protein product supports by *in vitro/in vivo* studies), PS4 (prevalence in affected individuals higher than controls, PP4 (patient’s phenotype highly specific for this condition) and PP5 (reputable sources report variant as pathogenic))	Confirmed the diagnosis of sickle cell anaemia as the most likely cause of the phenotype

* Prediction tools used: SIFT/LRT/Mutation Taster: D = damaging or deleterious, P = probably deleterious, B = benign, T = tolerated, N = neutral, A = disease causing (as per Mutation Taster), . = termination, - = no prediction.

** Classification as per ACMG guidelines; Class 5 = pathogenic; 4 = likely pathogenic; 3 = variant of uncertain significance (VUS); 2 = likely benign; 1 = benign.

AA, amino acid; AD, autosomal dominant; AR, autosomal recessive; CAPS, cryopyrin-associated periodic syndrome; HL, hearing loss; IP, inflammatory gene panel; LRT, likelihood ratio test; MAF, mean allele frequency; ROSAH, retinal dystrophy, optic nerve oedema, splenomegaly, anhidrosis and headache; SIFT, sorting intolerant from tolerant; VUS, variant of uncertain significance.

Case 2: *NLRP3* c.C2744G, p.(Thr915Arg) [*NLRP3* p.T915R] heterozygous: this is a novel variant, affecting the leucine-rich repeat (LRR) domain at the C-terminus of NLRP3, classified as a likely pathogenic as per ACMG guidelines. Parental testing confirmed the variant was *de novo*. On review, clinical features were consistent with cryopyrinopathy: episcleritis, papilloedema, bilateral SNHL, fatigue, arthralgia and systemic inflammation with elevated CRP (89 mg/l, (normal range 0–10 mg/l)), raised intracranial pressure (opening pressure 40 cm H_2_O, normal range 6–28 cm H_2_O)), and sterile CSF pleocytosis (15 leucocytes/mm^3^, no red cells; protein 0.35 g/l (reference range 0.10–0.50) glucose 3.3 mmol/l (reference range 2.5–4.5)).

Case 3: *ALPK1* c.A2204C p.(His735Pro) [*ALPK1* p.H735P] heterozygous: this is a novel missense variant in *ALPK1*, a gene recently associated with ROSAH syndrome: Retinal dystrophy, Optic nerve oedema, Splenomegaly, Anhidrosis and migraine Headaches [18]. This patient developed uveitis at 15.7 years, then bilateral SNHL, auditory distortion and vestibular symptoms 3 months later. He had mild renal involvement, with mildly raised renal tubular markers. This variant is currently of uncertain clinical significance but potentially highly relevant to the phenotype. Parental testing was recommended; however, the patient and family failed to return for any medical follow-up.

Case 4: *ADGRV1* c.G1849A p.(Val617Met) [*ADGRV1* p.V617M]; c.G3289A p.(Gly1097Ser) [*ADGRV1* p.G1097S]; c.A6994T p.(Ile2332Phe) [*ADGRV1* p.I2332F] heterozygous: variants in *ADGRV1*, classified as class 3 VUS. Variants in *ADGRV1*, encoding an adhesion G-protein coupled receptor 1 (ADGRV1), are associated with Usher syndrome type 2C, characterized by childhood-onset moderate SNHL, retinitis pigmentosa from adolescence and vestibular dysfunction [19, 20]. This case, with background of consanguinity and African ancestry, had bilateral uveitis, preceding unilateral SNHL in adolescence, and vestibular dysfunction. She presented with fever, headaches, elevated inflammatory markers, ANA positivity and immune-mediated glomerulonephritis on renal biopsy. Genetic testing was unable to establish if these variants are in *cis* or *trans*; next investigation would be parental testing to further inform pathogenicity. As reported in the literature, a diagnosis of Usher syndrome type 2C may be supported, if in *trans*, partially explaining her phenotype, although not uveitis.

Case 8: *MYO7A* c.1617dupC p.(Lys542Glnfs*5) [*MYO7A* p.K542Qfs*5] heterozygous, classified as pathogenic. Bi-allelic variants in *MYO7A* are associated with AR deafness and Usher syndrome type 1B and this variant reported in heterozygous form in a single previous report associated with deafness [21]. This patient presented in adolescence with interstitial keratitis, uveitis and bilateral SNHL, with auditory distortion and vestibular dysfunction, with low inflammatory markers. He had one other intronic variant in *MYO7A* c.3925-243A > G, thought to be benign. To further evaluate this finding, parental studies would be informative, but parental DNA was not available for us to elaborate further.

Case 10: *HBB* c.A20T p.(Glu7Val) [*HBB* p.E7V] homozygous: this is the most prevalent genotype associated with sickle cell anaemia (SCA) leading to haemoglobin S polymerization and erythrocyte sickling, and haemolysis. An inflammatory vasculopathy results from intra-vascular haemolysis [22]. This patient developed bilateral uveitis aged 6 followed by bilateral SNHL, vestibular dysfunction, headache, lymphadenopathy and raised inflammatory markers. She had bilateral labyrinthitis ossificans on neuroimaging, with suggestion of cochlear artery thrombus. This genotype confirms the diagnosis of SCA may be the likely cause of the phenotype, especially given MRI findings. This variant is classified as pathogenic (PS1, PS3, PS4, PP4 and PP5).

## Discussion

Through WES with a comprehensive genetic analytic workflow, a conservative estimate was that 30% (3/10) of our cohort of pCS had an alternative monogenic disease contributing to the phenotype based on presence of class 4/5 (likely pathogenic or pathogenic) genetic variants rather than true pCS. Two further patients had a possible genotype contributing to the phenotype, casting doubt on the clinical diagnosis of pCS. In the five patients without any suggestion of a monogenic diagnosis, the clinical diagnosis of pCS was more secure.

Case 2 had a novel likely pathogenic variant in *NLRP3* p.T915R, affecting the LRR region of NLRP3. Two groups have recently described individuals with Cryopyrin-associated periodic syndrome (CAPS) with variants affecting the LRR domain, in close proximity to the variant in this individual, with a distinct phenotype with more SNHL and headache and less urticarial rash, compared with classical CAPS associated with variants affecting the NACHT central nucleotide-binding oligomerization domain [23, 24]. This is in keeping with the phenotype of this case. Previously described functional studies on patients with pathogenic variants affecting the LRR domain, have shown elevated ASC (apoptosis-associated speck-like protein containing a CARD) concentration compared with classical CAPS patients and elevated IL-18 levels. The hypothesized pathophysiological mechanism is that a stimulating signal is required for full inflammasome activation, resulting in early and sustained IL-1β production [23]. Identification of this variant has implications for targeted treatment, with IL-1 blockade, and prognosis, with long-term follow-up for potential development of secondary amyloidosis. In light of this result, case 2 was trialled on canakinumab, which resulted in partial but incomplete improvement in headache and fatigue. Although canakinumab resulted in complete normalization of CRP, the patient reported that their joint symptoms seemed better controlled with tocilizumab and therefore chose to be converted back to that. It is well known that monoclonal antibodies do not penetrate the CNS well. This is also described in relation to patients with CAPS and CNS involvement, possibly explaining why the headache improved somewhat but not completely.

Case 3 had a novel rare variant, classified as a VUS, in *ALPK1* (p.H735P), associated with the recently described ROSAH syndrome. This describes a primarily inflammatory eye disease, including uveitis and, in some, as the only manifestation [25]. To date, SNHL has not been reported in ROSAH, although phenotypic heterogeneity is increasingly recognized. ALPK1 is a protein kinase, with a role in centrosome and ciliary function, and is an innate immune receptor for bacterial sugars [26]. Gain-of-function variants lead to an autoinflammatory syndrome caused by NF-κB-driven inflammasome activation, similar to A20 haploinsufficiency, Behçet’s disease and Blau syndrome, all associated with uveitis. Only five variants have been reported on Infevers, three of which are pathogenic/likely pathogenic (https://infevers.umai-montpellier.fr/web/) [accessed 2 February 2026]. This case failed to return for further medical review and therefore parental testing was not performed.

Patient 10 had SCA caused by a homozygous variant (*HBB* p.E7V) [27]. Uveitis is occasionally reported in individuals with SCA; and SNHL affects 13% of paediatric patients [28]. One published case report describes a Cogan’s phenotype, with SNHL, bilateral uveitis and raised inflammatory markers in a child with SCA [29]. Whether this case had an autoimmune CS superimposed on SCA, or alternatively, the full phenotype because of SCA is uncertain, although we suggest that the phenotype is most likely to be fully explained by SCA. The patient was managed with a 5-month course of corticosteroid only, without ongoing immunosuppression. Given that sickle cell anaemia is well described as an occasional cause of uveitis; and a rather common cause of sensorineural hearing loss in this population, almost certainly the result of sickling causing occlusive vasculopathy, it is unsurprising that sickle cell disease may masquerade as autoimmune Cogan’s syndrome in some patients. We therefore highlight this case so that clinicians are aware of this association.

Two patients had rare variants in genes associated with hearing loss: patient 8 *MYO7A;* and patient 4 *ADGRV1*, potentially significant since, if confirmed pathogenic, this would suggest glucocorticoids and immunosuppression would not be indicated for these patients. Compound heterozygous variants have been reported in *ADGRV1*, some with SNHL without other features of Usher syndrome and some associated with Usher syndrome 2C [30], including the combination p.V617M and p.I2332F (two of the three variants harboured by this individual) which has been previously reported in an individual with childhood-onset SNHL and Usher syndrome [31]. One other series reported an individual with compound heterozygous variants (p.I2332F and p.S5821P, classified as VUS) with Usher syndrome, with a wider phenotype including seizures and microcephaly [32].

Homozygous *MYO7A* p.K542Qfs*5 variants have been linked with hearing loss and Usher syndrome 1B, and this variant has been previously reported in a single affected Northern European case in heterozygous form (p.K542Qfs*5) [33–35]. However, in this study, the polyadenylation and promoter regions and enhancer elements were not interrogated for a second variant; it is plausible that this individual had a second variant, rather than the phenotype manifesting as a heterozygote [21]. This variant has been reported in two individuals from a UK cohort of Usher syndrome in combination with other pathogenic variants in *MYO7A* [35]. In some, there may be AD inheritance with variable genetic penetrance or digenic inheritance. This variant is therefore a class 5 pathogenic variant in heterozygous state for a predominantly AR condition. Parental studies would be informative, if the variant is *de novo*, this would add evidence to its significance, but a limitation is parental DNA was not available to us to elaborate further on this result.

These genetic findings have implications for the management of patients with the pCS phenotype; identification of alternative genetic autoinflammatory diagnoses facilitates more targeted treatment and more accurate prognostication and has implications for genetic testing of family members. Identification of alternative non-inflammatory genetic diagnoses associated with SNHL prevents unnecessary exposure of these patients to immunosuppressive treatments, which would not be expected to preserve or recover hearing loss. These findings are important for this rare group of patients and we advocate for genetic testing in individuals with the pCS phenotype.

There are several limitations to this study. Firstly, due to the rarity of this condition, the sample size is small, an issue pertinent to all rare disease research. This study was designed to identify if there are individuals with monogenic conditions mimicking CS, with potential implications on treatment of these individuals, and understanding of the phenotypic complexity, rather than to identify novel candidate genes, for which it is underpowered. There are limitations to our genetic analysis. Firstly, variants may have been missed that were not captured by our workflow. Additionally, there was limited access to subsequent family testing, and functional analysis, both of which could have contributed to further classification of variants. Finally, our study relied on short-read WES: non-coding regions of the genome, which were not sequenced, may harbour genes relevant to this cohort, and long-read technology would have facilitated phasing of variants and further variant classification.

In conclusion, clinicians should be cautious about conferring the lifelong diagnosis of pCS since 30% of our series had a plausible alternative monogenic explanation for the phenotype; and a further 2/10 cases had rare variants that could contribute the phenotype. We therefore suggest that paediatric patients presenting with features suggestive of CS undergo whole exome or whole genome sequencing, since genetic findings can direct more targeted treatment and prevent inappropriate immune suppression for some patients.

## Supplementary Material

keag436_Supplementary_Data

## Data Availability

The data underlying this article will be shared on reasonable request to the corresponding author.
