# Supplementary material for: Whole exome sequencing of paediatric patients with Cogan’s syndrome to identify monogenic mimics
Source: Rheumatology (Oxford). 2026 Aug 25;65(9):keag436. doi: 10.1093/rheumatology/keag436 (PMC13553075; doi:10.1093/rheumatology/keag436)
Supplement: Supplementary file 1 [file keag436_supplementary_data.docx]

**Supplementary Table S1: Customised hearing loss gene list which was included in the workflow**

| Gene | Mode of inheritance | Hereditary HearingLoss | PanelApp |
| --- | --- | --- | --- |
| *DIAPH1* | AD | Y | Y |
| *KCNQ4* | AD | Y | Y |
| *GJB3* | AD | Y | Y |
| *IFNLR1* | AD | Y | N |
| *GJB2* | AD, AR | Y | Y |
| *GJB6* | AD | Y | N |
| *MYH14* | AD | Y | Y |
| *CEACAM16* | AD, AR | Y | Y |
| *GSDME* | AD | Y | Y |
| *DNFNA5* | AD | Y | Y |
| *WFS1* | AD | Y | Y |
| *LMX1A* | AD | Y | Y |
| *TECTA* | AD, AR | Y | Y |
| *COCH* | AD, AR | Y | Y |
| *EYA4* | AD | Y | Y |
| *MYO7A* | AD, AR | Y | Y |
| *COL11A2* | AD, AR | Y | N |
| *POU4F3* | AD | Y | Y |
| *MYH9* | AD | Y | Y |
| *ACTG1* | AD | Y | Y |
| *MYO6* | AD, AR | Y | Y |
| *SIX1* | AD | Y | Y |
| *SLC17A8* | AD | Y | Y |
| *REST* | AD | Y | N |
| *GRHL2* | AD | Y | Y |
| *NLRP3* | AD | Y | N |
| *TMC1* | AD, AR | Y | Y |
| *COL11A1* | AD | Y | Y |
| *CRYM* | AD | Y | Y |
| *P2RX2* | AD | Y | Y |
| *CCDC50* | AD | Y | Y |
| *MIRN96* | AD | Y | N |
| *TJP2* | AD | Y | N |
| *TNC* | AD | Y | N |
| *SMAC* | AD | Y | N |
| *DIABLO* | AD | Y | N |
| *TBC1D24* | AD, AR | Y | Y |
| *CD164* | AD | Y | N |
| *OSBPL2* | AD | Y | Y |
| *HOMER2* | AD | Y | N |
| *KITLG* | AD | Y | N |
| *MCM2* | AD | Y | N |
| *PTPRQ* | AD, AR | Y | Y |
| *DMXL2* | AD, AR | Y | Y |
| *MYO3A* | AD, AR | Y | Y |
| *PDE1C* | AD | Y | N |
| *TRRAP* | AD | Y | N |
| *PLS1* | AD | Y | Y |
| *ATP2B2* | AD | Y | N |
| *SCD5* | AD | Y | N |
| *SLC12A2* | AD | Y | Y |
| *MAP1B* | AD | Y | N |
| *RIPOR2* | AD, AR | Y | N |
| *FAM65B* | AD, AR | Y | N |
| *MYO15A* | AR | Y | Y |
| *SLC26A4* | AR | Y | Y |
| *TMIE* | AR | Y | Y |
| *TMPRSS3* | AR | Y | Y |
| *OTOF* | AR | Y | Y |
| *CDH23* | AR | Y | Y |
| *GIPC3* | AR | Y | Y |
| *STRC* | AR | Y | Y |
| *USH1C* | AR | Y | Y |
| *OTOG* | AR | Y | Y |
| *OTOA* | AR | Y | Y |
| *PCDH15* | AR | Y | Y |
| *RDX* | AR | Y | Y |
| *GRXCR1* | AR | Y | Y |
| *GAB1* | AR | Y | N |
| *TRIOBP* | AR | Y | Y |
| *CLDN14* | AR | Y | Y |
| *WHRN* | AR | Y | Y |
| *CDC14A* | AR | Y | Y |
| *ESRRB* | AR | Y | Y |
| *ESPN* | AR | Y | Y |
| *HGF* | AR | Y | N |
| *ILDR1* | AR | Y | Y |
| *ADCY1* | AR | Y | N |
| *CIB2* | AR | Y | Y |
| *MARVELD2* | AR | Y | Y |
| *BDP1* | AR | Y | N |
| *PDZD7* | AR | Y | Y |
| *PJVK* | AR | Y | N |
| *SLC22A4* | AR | Y | N |
| *SLC26A5* | AR | Y | Y |
| *LRTOMT* | AR | Y | Y |
| *COMT2* | AR | Y | Y |
| *DCDC2* | AR | Y | N |
| *LHFPL5* | AR | Y | Y |
| *S1PR2* | AR | Y | Y |
| *PNPT1* | AR | Y | Y |
| *BSND* | AR | Y | Y |
| *MSRB3* | AR | Y | Y |
| *SYNE4* | AR | Y | Y |
| *LOXHD1* | AR | Y | Y |
| *TPRN* | AR | Y | Y |
| *GPSM2* | AR | Y | Y |
| *OTOGL* | AR | Y | Y |
| *ELMOD3* | AR | Y | N |
| *KARS* | AR | Y | Y |
| *SERPINB6* | AR | Y | Y |
| *CABP2* | AR | Y | Y |
| *NARS2* | AR | Y | N |
| *MET* | AR | Y | N |
| *TSPEAR* | AR | Y | N |
| *TMEM132E* | AR | Y | N |
| *PPIP5K2* | AR | Y | N |
| *GRXCR2* | AR | Y | N |
| *EPS8* | AR | Y | Y |
| *CLIC5* | AR | Y | N |
| *DFNB32* | AR | Y | N |
| *EPS8L2* | AR | Y | Y |
| *ROR1* | AR | Y | N |
| *WBP2* | AR | Y | N |
| *ESRP1* | AR | Y | N |
| *MPZL2* | AR | Y | Y |
| *GRAP* | AR | Y | N |
| *SPNS2* | AR | Y | N |
| *CLDN9* | AR | Y | Y |
| *CLRN2* | AR | Y | N |
| *GAS2* | AR | Y | N |
| *PRPS1* | X-linked | Y | Y |
| *POU3F4* | X-linked | Y | Y |
| *SMPX* | X-linked | Y | Y |
| *AIFM1* | X-linked | Y | N |
| *COL4A6* | X-linked | Y | N |
| *ABHD12* | AR | N | Y |
| *ADGRV1* | AR | N | Y |
| *ALMS1* | AR | N | Y |
| *ATP6V1B1* | AR | N | Y |
| *BCS1L* | AR | N | Y |
| *CEP250* | AR | N | Y |
| *CEP78* | AR | N | Y |
| *CLRN1* | AR | N | Y |
| *COL2A1* | AD | N | Y |
| *COL4A5* | X-linked dominant | N | Y |
| *COL9A1* | AR | N | Y |
| *COL9A2* | AR | N | Y |
| *DFNB59* | AR | N | Y |
| *DNMT1* | AD | N | Y |
| *FDXR* | AR | N | Y |
| *FOXI1* | AR | N | Y |
| *GATA3* | AD | N | Y |
| *HARS2* | AR | N | Y |
| *KCNJ10* | AR | N | Y |
| *MT-RNR1* | Mitochondrial | N | Y |
| *MT-TS1* | Mitochondrial | N | Y |
| *RNF220* | AR | N | Y |
| *SGPL1* | AR | N | Y |
| *SLC52A2* | AR | N | Y |
| *SPATA5L1* | AR | N | Y |
| *USH1G* | AR | N | Y |
| *USH2A* | AR | N | Y |
| *USP48* | AD | N | Y |
